# Book Review: The Routledge Handbook of Cognitive Linguistics (1st Edition)

**DOI:** 10.3389/fpsyg.2021.697145

**Published:** 2021-05-31

**Authors:** Yang Yao, Qiujun Su

**Affiliations:** Faculty of Foreign Languages, Southwest Forestry University, Kunming, China

**Keywords:** cognition, cognitive linguistics, the Routledge handbook, book review, linguistics

## Introduction

Cognitive linguistics is a branch of linguistics. Based on the theory of the second generation of cognitive science and experiential philosophy, it was born on the basis of opposing the transformational generative grammar of mainstream linguistics and began to take shape from the late 1980s to the 1990s. Cognitive linguistics involves artificial intelligence, linguistics, psychology, systems theory, and other disciplines. Aiming at the innate view held by generative linguistics, it proposes that the creation, learning and application of language must be explained through human cognition, because cognitive ability is the foundation of human knowledge.

As a relatively new research paradigm in linguistic studies, Cognitive Linguistics has made a lot of remarkable achievements both in theory and in practice in the past 40 years. It has been widely used and become a multidisciplinary, interdisciplinary, and transdisciplinary school of linguistics. Cognitive Linguistics is closely related to psycholinguistics and systemic functional linguistics. It is based on cognitive science, and the research of cognitive linguistics is to use cognitive psychology and linguistic theory to study the relationship between representation, concept, and language structure. Psycholinguistics is based on psychology, it involves language memory, phonological perception, language learning, and so on. Compared with Systemic Functional Linguistics, Cognitive Linguistics focuses more on the internal factors of the speaker, that is, the psychological mechanism and the generation and exchange of meaning in the cognitive process. Systemic Functional Linguistics focuses on the social communication of language, and studies language from the social function and use situation of language.

*The Routledge Handbook of cognitive linguistics* gives a comprehensive introduction to cognitive linguistics. It covers the key areas of cognitive linguistics and contains a wide range of perspectives and methods in this area (Wen and Taylor, 2021). The book is worthy of serious study by cognitive linguistics learners with its cutting-edge views and reasonable suggestions.

## The Book

This book contains a collection of works in Cognitive Linguistics. A serial of profound, systematic, and internationally oriented studies written by international specialists in the field has been presented in this book. The book contains four parts (43 chapters).

The first part of the book covers basic theories and hypotheses. This is the most important chapter in which readers can understand the basic concepts and historical developments of cognitive linguistics, including cognitive semantics, frame semantics, natural semantic metalanguage, along with cognitive grammar, construction grammar, and word grammar.

Part 2 collects the most popular topics in cognitive linguisticsis, mainly about cognitive pragmatics, cognitive poetics, metaphor, metonymy, humor, construal, iconicity, and image schemas, categorization, embodiment, motivation, constructionalization, intersubjectivity, grounding, multimodality, and linguistic synaesthesia, and so on. This chapter presents the most mainstream research of cognitive linguistics for researchers.

Part 3 of the book focuses on relationships and interfaces between cognitive linguistics and other areas, including figurative language, gesture, signed languages, cultural linguistics, language acquisition and pedagogy, linguistic typology, digital lexicography, and translation studies. It shows the integration of cognitive linguistics and other research fields or disciplines.

For students and young researchers, the last chapter should not be missed. Studies in this chapter look to new horizons in cognitive linguistics, presenting a series of approaches to social, diachronic, neuroscientific, biological, ecological, multimodal, and quantitative studies. Following the pace of these studies, we can further explore and develop the research of cognitive linguistics.

In sum, this book introduces the latest development of cognitive linguistics and predicts its future development. It makes a rational analysis of the criticisms of other schools on cognitive linguistics, and puts forward the direction of improvement and efforts. The book has its own characteristics compared with other books on cognitive linguistics. Some books in this area only introduce or summarize the results of previous studies. Researchers in this book not only analyze the previous theories in detail and systematically, but also express their own views and point out the development direction of cognitive linguistics.

## Comments

Cognitive linguistics is not a single language theory, but represents a research paradigm. It holds that people's daily experience is regarded as the basis of language use, and it focuses on explaining the inseparable relationship between language and general cognitive ability. Although these linguistic theories are different, their basic assumptions about language are almost the same. They all agree with the basic viewpoints, but differ in the specific linguistic phenomena discussed and concerned.

Unfortunately, most of the articles in this book are still using traditional western research frameworks, methods and corpus, so its interpretation tends to have certain western contextual background and characteristics. Studies of Cognitive Linguistics still has a long way to go in China. Looking forward to the development of cognitive linguistics, Chinese scholars should make full use of the advantages of Chinese, Chinese dialects and the diversity of minority languages in China, fully describe and compare the similarities and differences of these languages and dialects and their causes, so as to provide unique and richer evidence for revealing the influence of language on cognitive style and cultural development. At the same time, through the theory of cognitive linguistics, we can deeply explore the cognitive style and cultural background behind Chinese grammar, truly reveal the characteristics of Chinese grammar and pragmatics, and make contributions to the theory of general linguistics.

Throughout the development of Cognitive Linguistics in the past 40 years, it has achieved remarkable achievements both in theory and practice. With the development of cognitive linguistics, the core concepts of category theory, metaphor/metonymy theory, cognitive semantics, and construction grammar have made great progress. At the same time, it also integrates with other disciplines, such as neurolinguistics, psycholinguistics, sociolinguistics and philosophy, and promotes each other's development. In addition, how to use the theory to better understand the process of second language acquisition and effectively carry out language teaching activities has become the focus of scholars' discussion in recent years. But at the same time, this book, as well as Cognitive Linguistics, is still worthy of improvement. Studies in this book, as well as many other Cognitive Linguistic studies, often focused on introspective research methods and restricts itself to oral language studies, many theoretical hypotheses are put forward but fewer empirical studies have been done. Individual differences and social factors in language are often ignored.

However, *The Routledge Handbook of Cognitive Linguistics* still provides the most up-to-date research on Cognitive Linguistics. The book describes a systematic research paradigm and comprehensively interpret Cognitive Linguistic studies from an international perspective. It offers a starting point of studies in this area, and provides new frameworks for analyzing and interpreting languages. This book will be a vital resource and a very thought-provoking reference for all students and researchers working in this area.

## Author Contributions

YY has written the book review. QS was YY's colleague, he helped to examine and modify the review. All authors contributed to the article and approved the submitted version.

## Conflict of Interest

The authors declare that the research was conducted in the absence of any commercial or financial relationships that could be construed as a potential conflict of interest.
